# Supplementary material for: An Intranuclear Sodalis-Like Symbiont and Spiroplasma Coinfect the Carrot Psyllid, Bactericera trigonica (Hemiptera, Psylloidea)
Source: Microorganisms. 2020 May 8;8(5):692. doi: 10.3390/microorganisms8050692 (PMC7284866; doi:10.3390/microorganisms8050692)
Supplement: Supplementary file 1 [file microorganisms-08-00692-s001.zip › microorganisms-779811-supplementary.docx]

**Supplementary Materials:**


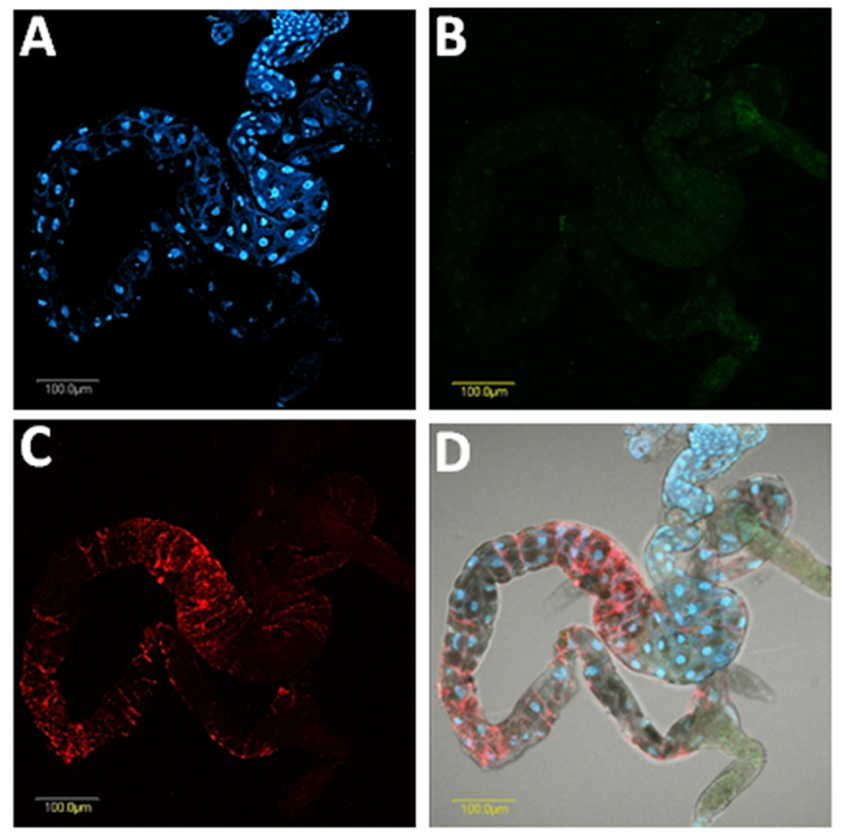


**Figure S1.** Localization of *Sodalis* and CLso using FISH with specific probes in BT midgut tissue 10 days after CLso acquisition. A, DAPI staining (blue) of BT midgut nuclei. B, Localization of *Sodalis* (green) in the same midgut seen in (A) but showing very faint endonuclear localization. C, Localization of CLso (red) in the same midgut seen in A and B. D, Overlay of *Sodalis*, CLso and midgut nuclei under bright field.
